# PD59 Review Of The International Epidemiology Of Long COVID

**DOI:** 10.1017/S0266462324003179

**Published:** 2025-01-07

**Authors:** Michelle Norris, Fearghal Comaskey, Carol Mc Loughlin, Niamh Merriman, Barrie Tyner, Aislinn O’Mahony, Marie Carrigan, Patricia Harrington, Máirín Ryan, Michelle O’ Neill

## Abstract

**Introduction:**

Approximately 65 million people worldwide have Long COVID. Long COVID is a complex condition with more than 200 symptoms, which can substantially affect the lives of individuals. The evidence base for Long COVID is evolving rapidly and, therefore, an up-to-date understanding of the prevalence and risk factors of Long COVID is necessary to inform service delivery and allocation of healthcare resources.

**Methods:**

A systematic literature review was conducted. Long COVID epidemiological literature published after October 2021 was identified in the MEDLINE, Embase, and Cochrane Library databases. Data extraction and quality appraisal were completed by one reviewer and checked for accuracy and omissions by a second reviewer. The following subgroups of interest were identified: general population; children and older adults; individuals who are medically vulnerable; and individuals with a history of severe COVID-19. Narrative synthesis of the prevalence and symptoms of Long COVID and of risk factors associated with the development of Long COVID was conducted.

**Results:**

Over 3,000 documents were identified, of which 51 primary research studies met the inclusion criteria and were deemed of fair or good quality. Long COVID prevalence estimates ranged from 1.8 to 53.1 percent in the general population; 0.1 to 65.7 percent in children; 5.6 to 80.8 percent in older adults; 12.4 to 29.7 percent in medically vulnerable individuals; and 9.8 to 94.6 percent in individuals with a history of severe COVID-19. A wide range of symptoms were identified, with fatigue and neurological and respiratory symptoms being commonly reported. Female sex and increased age were identified as risk factors for developing Long COVID.

**Conclusions:**

Long COVID is a complex condition involving a wide range of symptoms, which may result in significant reductions in quality of life and functioning in some individuals, a substantial burden on healthcare systems, and broader economic impacts. In planning healthcare delivery for this population, a focus on multidisciplinary holistic care will be necessary.

